# Connection in Youth Development Key to the Mental Health Continuum in Ghana: A Structural Equation Model of Thriving and Flourishing Indicators

**DOI:** 10.3389/fpsyg.2021.676376

**Published:** 2021-10-21

**Authors:** Russell Sarwar Kabir, David Teye Doku, Nora Wiium

**Affiliations:** ^1^Graduate School of Humanities and Social Sciences, Hiroshima University, Higashihiroshima, Japan; ^2^Department of Population and Health, University of Cape Coast, Cape Coast, Ghana; ^3^Department of Psychosocial Science, Faculty of Psychology, University of Bergen, Bergen, Norway

**Keywords:** mental health, flourishing, five Cs of PYD, youth and emerging adults, Ghana, psychosocial support

## Abstract

Practitioners from sub-Saharan Africa are working to provide evidence-based intervention programs to address the mental health of established adults in poor rural communities in Ghana. However, institutions in Ghana also pursue youth policy for training human capital that can contribute to national development as a strategy to leverage its heavy demographic makeup of adolescents and emerging adults. Positive Youth Development (PYD) is a framework for measuring indicators of thriving for such youthful populations. Studies have recently examined PYD in terms of developmental assets with mental illness, but less is known about their interaction with the continuum of mental health, which poses strength-based theoretical distinctions about the conditions of human flourishing. Investigating positive mental health in terms of well-being, along with developmental indicators from another conception of PYD with strong theoretical grounding known as the 5Cs, represents a salient cross-section of Ghana’s current trajectory along these policies and evaluations of culturally attuned well-being toward youth-focused efforts. Thus, the aim of this study was to clarify whether developmental constructs could predict positive mental health outcomes for indications of adaptive regulation processes and cultural concepts of well-being. We used structural equation modeling of the PYD domains (i.e., the 5Cs) to provide novel insights into individual differences in factors of thriving with flourishing-languishing indicators from the mental health continuum (MHC; i.e., factors of *Emotional*, *Social*, and *Psychological Well-being*) for 710 youth and emerging adults (*M* age=19.97, *SD*=1.93) attending a university in Ghana. The results showed supported paths for *Connection*, which was associated with all three MHC well-being domains (*β*s=0.34–0.41), and *Caring*, which was associated with *Psychological Well-being* (*β*=0.27), as factors to consider for youth who are expected to underwrite Ghana’s development under economically challenged conditions. These findings support evidence-based program outcomes and prior work that situates social relations as a key route to maintaining well-being, advancing research on the specificity of predictors for positive mental health factors among young people in an enterprising Ghana.

## Introduction

Positive Youth Development (PYD) is a highly scrutinized framework for classifying dimensions in child and adolescent progress toward becoming productive members of society (Larson, 2000; Lerner et al., 2018). PYD emphasizes a strength-based system of constructs, where youth and emerging adults possess and enact skills relevant to the cultivation of themselves, their communities, and their societies. A review of current perspectives on the field of PYD suggested five key overarching frameworks for theorizing and conceptualization: Benson’s developmental assets, Catalano’s 15 PYD constructs (Catalano et al., 2012), the “being” perspective of character and spirituality, social and emotional learning (SEL) approaches, and Lerner’s 5Cs of PYD (Shek et al., 2019). Throughout these frameworks, the interplay between personal and contextual resources is especially linked to a process of *adaptive developmental regulation*, to which thriving youth make positive contributions to their spheres in ways that garner upstream effects in their transition to adulthood (Hamilton et al., 2004; Benson et al., 2006).

Studies have begun examining PYD and mental illness across cultural contexts, such as associations between substance abuse among Latin American college students (Manrique-Millones et al., 2021), prolonged sadness and suicide attempts among high school students in Norway (Wiium et al., 2021), and life satisfaction and hopelessness among mainland Chinese adolescents (Zhou et al., 2020). Subjective well-being has been described as a consequence of the PYD attributes (Martela and Sheldon, 2019), but gaps remain on the state of explicit relationships between developmental resources and socioemotional well-being (Valois, 2014; Zhou et al., 2020). A validation study by Kennes et al. (2020) suggested that promoting positive emotions, supportive parent-adolescent relationships, and supportive school environments might foster protective factors from development to adolescent well-being, but to the best of our knowledge, studies have not formally investigated the 5Cs of PYD and *positive* mental health. This paper models the relationships between the 5Cs of PYD as resources and competencies related to “thriving” with the Mental Health Continuum (MHC; Keyes, 2002) as indicators of well-being in terms of “flourishing” for a cross-section of the markers of adaptive developmental regulation among the illustrative, underrepresented, and potentially idiosyncratic, target population of Ghanaian youth.

The widely operationalized conceptualization of the framework known as the “5Cs of PYD” includes developmental outcomes related to *Character* (having integrity, moral commitment, and respect for societal and cultural rules), *Confidence* (having a sense of mastery and future, positive identity, and self-efficacy), *Connection* (having healthy relation to friends, family, school, and community), *Caring* (showing empathy and sympathy), and *Competence* (in academic, social, and vocational skills). The 5Cs of PYD have been validated in numerous contexts typically estimated as components in a five-factor model (Geldhof et al., 2014), although some psychometric challenges have been noted (Chen et al., 2018). PYD is a framework that comprehensively captures key components of interest to youth interventions, but more research on implementation science and PYD is required to match its specificity to interventions with greater clarity (Eichas et al., 2019).

Previous research on PYD in Ghana has been useful toward advancing progress along sustainable development goals, namely, by identifying missing external assets with emerging adults (Wiium, 2017), gender norms and socialization practices among adolescents (Wiium et al., 2019), and tendencies in environmental concerns among university students (Kabir and Wiium, 2021). Due to the linkages of overlapping categories of well-being in the Ghanaian context, there is a need to refine theoretical assumptions of well-being with prevailing contextual needs (Fadiji et al., 2019). Modeling the relationships between the MHC and PYD as coarse-grained markers of psychological, social, and emotional functioning on the ground could clarify areas to target for resources, program efforts, or countermeasures, add explanatory value to their mechanism of effects, and provide another source of analysis to understand the multidimensional and culturally shaped nature of well-being.

### Global Trends in Considerations for Well-Being and Positive Mental Health

Mental health and psychosocial support services (MHPSS) are included in the scope and major programs of work at premier international organizations, where well-being is used to track the progress of societies along important areas of institutional, community, and individual development. In line with verbal theories in global research trends on well-being, many international efforts have targeted and tracked changes using the taxonomy of hedonic and eudaimonic well-being as indicators. Specifically, *subjective well-being* has successfully been measured as a cross-cultural target by international institutions (OECD, 2013), but not without scrutiny that calls for emic-etic agreement with local concepts (Rappleye et al., 2020). Despite other critical measurement issues and ambiguities about operationalization (Disabato et al., 2016; Linton et al., 2016), and broader debates on the role of socioeconomic indicators like sensitivity to changes in living conditions (e.g., Nakazato et al., 2011), genetic correlations support overlap for hedonic and eudaimonic well-being (Baselmans and Bartels, 2018), and progress in positive neuroscience has delineated the structural and functional neural foundations for positive human functioning (Kong et al., 2020), identifying the role of the precuneus (Sato et al., 2015), in favor of construct representation. Matching well-being dimensions with adolescent outcomes has also been specified for interpretations about positive and negative mental health in emerging adulthood, which emphasize mental health promotion (Winzer et al., 2014; Winzer, 2019).

In keeping with globally recognized efforts to promote mental health and bestow an operational definition that endorses holism, positive mental health has been formulated as being conceptually related to but categorically distinct from the constellations of symptoms and constituents that predicate mental illness (Keyes et al., 2008). This distinction stems from the logical supposition that the presence or absence of mental illness itself does not sufficiently satisfy the necessary condition(s) that would contain the presence or absence of mental health. Empirical observations with extensive and detailed validity matrices offer support for this position and provide an additional axis of mental health to the *de facto* axis of deficit-based medical models of psychopathology constituting “mental illness.” The resultant dual-axis model of mental health harbors strengths in specificity matching by providing grounded insights in indicators, such as the number of days of depression reported by adults in different and longitudinal waves of the Midlife Development in the United States (MIDUS) study (Keyes, 2002; Keyes et al., 2002) and others in the North American context (Keyes, 2007; Keyes et al., 2020), as well as in construct-identifying research with populations from South Africa (Keyes et al., 2008).

This “two continua” model proposes a scale that typically arbitrates two or three levels of categorical well-being that emerge from a range of indicators and can be described as an axis of languishing, to possessing moderate levels of mental health, to flourishing (Luijten et al., 2019; Winzer, 2019). This view represents the “flourishing” turn that constitutes a foundation in positive psychology and harmonizes with Ryff’s influential underpinnings for theorizing eudaimonic sources of well-being in addition to hedonic ones (Keyes et al., 2002). A short form instrument that spans the nomological network of eudaimonic and hedonic well-being was developed and found to match these theoretical constraints, known as the Mental Health Continuum-Short Form (MHC-SF; Keyes et al., 2008; Westerhof and Keyes, 2010; Luijten et al., 2019). The MHC-SF has shown high degrees of psychometric quality (Lamers et al., 2011) and has extended to contexts outside of its nation and language of origin as a scale (Luijten et al., 2019), such as Netherlands (Lamers et al., 2011), Italy (Petrillo et al., 2015), and Egypt (Salama-Younes, 2011). Additionally, detailed psychometric investigations for the MHC have been conducted in the context of students in the United States and young adults in Korea (Joshanloo, 2017, 2020), with adolescents in Asian countries (Lim, 2014; Guo et al., 2015), Dutch-speaking adolescents (Kennes et al., 2020), in Ghana (Appiah et al., 2020b), and across cultural groups (Joshanloo et al., 2013). Overall, efforts to establish the two-continua model and MHC-SF suggest that studies measuring public mental health prioritize and integrate psychological symptoms with an inventory of client strengths or assets as they align with the changing paradigm of helping, provide a better picture of functioning, and link to internationally agreed-upon advances in the standards and definitions of mental health and well-being (Kobau et al., 2011; Winzer, 2019; Keyes et al., 2020).

### The Country and Context of Ghana: An Illustrative Case of Culturally Shaped Psychological Makeup for Well-Being and Tracked Incentives for Policy Mapping

Ghana is a rapidly changing country in terms of demography and diversification of industry. Youth and emerging adults make up a large percentage of the total population, with reports estimating as much as 65 percent (United Nations, 2015), which makes the tracking of developmental outcomes and characteristics of youth in Ghana one with longitudinal significance by providing a barometer of local and national progress. Areas of special interest to strategic youth policy include Education and Skills Training, Youth and Employment Mentoring; Sports and Recreation; and Youth, Patriotism and Volunteerism (Ghana Ministry of Youth and Sports, 2010), which have been prioritized to ensure that Ghanaian youth acquire the skills and competencies needed to contribute to nationwide endeavors. Many young people in Ghana come from agrarian, traditional, and collectivistic households as first-time university-goers with high kinship expectations for sharing the profits of productive earning. This culturally shaped psychological makeup, in tandem with a developing mental health infrastructure that could constitute a “treatment gap” from a lack of capacity to provide MHPSS relative to need (World Health Organization, 2017), motivates a study of mental health indicators in the context of Ghana. In this way, youth and emerging adults represent a marker of the success of strategic youth policy as their tendencies denote a platform for needs identification and constraint satisfaction under the circumstances of growth across sectors.

Translating global level studies into local practice in sub-Saharan Africa research teams in Ghana have taken steps to address this gap by providing descriptions of the prevalence of positive mental health in clinical populations and both developing and implementing a comprehensive evidence-based intervention to address mental health in rural and poor communities. In a random sample of 62 adult patients with sickle cell disease, Appiah et al. (2020a) found higher levels of positive mental health with the MHC, such that 66% were flourishing, 26% were moderately mentally healthy, and 8% were languishing, suggesting that interventions for positive mental health could serve as a buffer to psychopathological risk. In a series of implementation studies to develop such a program, a community-based multicomponent positive psychology intervention for mental health known as the *Inspired Life Program* (ILP) was developed by Appiah et al. (2020b), with pre-test prevalence percentages of 25% flourishing in the intervention group (21.4% in the control group), 27.5% moderately mentally healthy in the intervention group (54.8% in the control group), and 47.5% languishing in the intervention group (23.8% in the control group). As a result of the 10-session intervention which reported medium to large effect sizes for MHC domains, 77.5% of the participants in the intervention group were flourishing compared to 38.1% for the control group at the 3-month follow-up, showing that the gains in the primary outcome of positive mental health were maintained in the period after the intervention. Although the control group was conditioned as an assessment only group rather than treatment as usual, the community-based participatory research approach to the development and administration of the ILP showed many indicators of implementation success marked by clear effect sizes, practical relevance, and guidance for practitioners (Appiah et al., 2021a). As these studies utilizing the MHC in Ghana focused on known groups and communities, they made efforts to maximize demographic representativeness in their study designs; however, their focus was not on youthful populations and reported chiefly on established and elderly adults. Overall, these studies provide a precedent for mental health implementation and foundation for the study of the MHC constructs in Ghana.

The core research question of the present study on whether young people who are thriving are also flourishing aims to build on prior studies that have clarified the characteristics of well-being in the Ghana context. This investigation not only has the potential to clarify the appropriate context of PYD interventions (Eichas et al., 2019), but also the focus of mental health implementation programs in Ghana, such as the ILP. The ILP is notably one of the first programs of its kind to feature a sample drawn from a collectivistic society (Appiah et al., 2020b). Discussion of the ILP suggested that the high level of interactivity and collectivistic nature of Ghanaian people might have contributed to the success of the community-based program, observing that “high enthusiasm and participants’ inclination to share their personal experiences with others,” (Appiah et al., 2020b, p. 852) indicating an opportunity self-reflection and self-disclosure. Another possibility is the way that well-being factors are conceived by people in Ghana. In a cultural model of the words for “well-being” in four Ghanaian languages, a cognitive-semantics-based interpretation across them supported etic conceptions for “good health” and “positive affective states” (Osei-Tutu et al., 2020), suggesting relationships to *Emotional Well-Being*. Addai et al. (2014) also found self-rated health status as a supported factor in a study of well-being in Ghana. However, elements of “good living,” comprised of “moral living,” “material success,” and “proper relationality” as facets, and an emic conception of “peace of mind” was also posited as possibly missing notions in efforts to quantify well-being (Osei-Tutu et al., 2020). These concepts taken inductively suggest that the PYD domains of *Caring* and *Character* might provide paths to the MHC for youth in Ghana.

Such local concepts also link to concerns that the *de facto* (or “hegemonic” psychological science) frameworks of well-being used in international assessment possess measurement biases might reflect preferences for Western-style individualism that overlook an “interdependent” dimension of contentment as an ingredient to living well (Rappleye et al., 2020). Interdependence is supported in the case of studies with individuals in Ghana who engaged in social participation (Amoah, 2018), which emerged as a significant predictor of health literacy and positive peer relations (Avogo, 2013; Wilson and Somhlaba, 2016). In Afrocentric conceptions of the axis of individualism–collectivism, a dialectic pattern of independent and interdependent orientations has been proposed to explain their relational contributions to finding meaning in life (Wissing et al., 2020). Social engagement has also been tied to the matter of religious behavior as a standout reason for the cultural shaping of the Ghanaian psychological makeup, to the degree that religious variables predicted well-being at the individual level (Addai et al., 2014). Longitudinally, the quality of relationships in the context of parental care was a major factor in child well-being for non-migrant families (Cebotari et al., 2018), and unhappiness was associated with parental neglect among independent child migrants (Amoah, 2020). Overall, well-being in Ghana is characterized by collective interactions and the support from others (Avogo, 2013), in ways that would suggest that well-being factors might relate to or prioritize to social competencies in the 5Cs, such as *Competence* and *Connection* for youth in Ghana.

In contrast to the salubriousness of collectivism, however, is the notion that some combinations of family duties and economic pressures also present push factors that might cascade into negative psychological states, such as social isolation and life unpredictability or lowly endorsable positive well-being. These suggest that the development of self-reliance as a competency might play an important role in holistic functioning, and thus, a role for *Confidence* in the developmental domain of PYD and its MHC counterpart of *Psychological Well-being*. In one example, qualitative evaluations suggested that realizing goals and showing competence in the face of adversity as indicative of autonomy could be explained as a key route to well-being (Amoah, 2020). Additionally, in studies on thriving in sub-Saharan African contexts by Adams et al. (2018), academic performance and achievement motivation were pointed out as key cross-cutting and global factors, which are marked by their individualistic focus, ultimately showing that internal assets were the only indicators associated with academic performance from supported path coefficients. In another contrast shown in Wiium (2017), “support” as defined by developmental assets was a factor that many Ghanaian survey participants reported a tendency *not* to experience, suggesting a lack of perceived external assets despite growing up in a cultural context characterized by collectivism. Finally, in the case of high school and university students in Ghana, a small but deleterious effect to psychological well-being was reported in a study on exposure to cyberbullying (Sam et al., 2019), suggesting that the school environment could affect peer relations as a form of social well-being. Together, the developmental context of emerging adults in Ghana represents an informative case of relationships to examine types of well-being, their status, and characteristics, and whether thriving emerging adults exhibit a profile of these tendencies.

### The Present Study: A Snapshot of Thriving and Flourishing for Youth and Emerging Adults in Ghana

This study is motivated to clarify the fit and relationships of global and local measurement strategies for well-being and developmental attributes of youth experiencing growth in Ghana. Our study structures these relationships with the measurement tool and framework of the domains of “thriving” from the 5Cs of positive youth development (Lerner et al., 2005) as they match up with the mental health continuum (Keyes et al., 2008) for tendencies in emotional, social, and psychological well-being as “flourishing” indicators.

#### Research Question 1: Do the PYD Domains Predict Positive Mental Health?

As both theories of thriving and flourishing are characterized by positive psychological outcomes, the direction of relationships was expected to be positive for all factors. Thus, high scores on the 5Cs of PYD were hypothesized to associate with high scores on the MHC (Hypothesis 1).

#### Research Question 2: Does Each PYD Domain Predict a Pattern of Mental Continuum Factors?

The magnitude of relationships between factors was expected to differ by PYD domain due to the scope of constructs for each framework. Specifically, *Confidence* was expected to associate strongly with *Psychological Well-being* due to internal characteristics in their shared factor operationalizations rooted in self-esteem and self-worth (Hypothesis 2). *Character* and *Caring* were expected to cut across all MHC types of well-being (to include *Emotional Well-being* as a source of positive affective states) due to their shared basis in values and concern for others (Hypothesis 3). In light of considerations for the measurement of well-being that do not sufficiently cover factors of social relations, the components of the 5Cs that handle social competencies (*Competence* and *Connection*) and the MHC-SF factor of *Social Well-being* were expected to associate due to the influences of the interdependent cultural makeup of Ghana (Hypothesis 4).

These hypothesized relationships were used to examine whether developmental constructs could predict positive mental health outcomes for indications of adaptive regulation processes and provide insights into cultural concepts of well-being.

## Materials and Methods

### Study Participants

A comprehensive set of psychometric instruments was given to youth and emerging adults in the University of Cape Coast, Ghana to investigate relationships in the sample population. A total of 723 first year university students (52% females) responded to the questionnaire. The age range of the full sample was 15–30, with a median age of 20 and a mean age of 20.04. Participants were selected through convenience sampling. The highest level of education was postsecondary for fathers (44%) and mothers (29%).

### Measures

#### Positive Youth Development – Short Form

The PYD-SF is a 34-item scale validated across numerous cross-national contexts (Tirrell et al., 2019). The PYD-SF measures thriving indicators with sample items for each of the Cs (*Competence*, *Confidence*, *Character*, *Connection*, and *Caring*), such as, “I have a lot of friends,” “I really like the way I look,” “I usually act the way I am supposed to,” “I am a useful and important member of my family,” and “It bothers me when bad things happen to any person,” respectively. Participants responded to a five-point Likert scale that ranges from 1 (*Strongly Disagree*) to 5 (*Strongly Agree*), to which high factor scores indicate high presence of each of the Cs. Using the robust maximum likelihood estimator, a measurement model of the 5Cs was previously identified in prior work with university students in Ghana with the same set of items (Geldhof et al., 2014; Kabir and Wiium, 2021).

#### Mental Health Continuum-Short Form

The MHC-SF is a 14-item instrument whose three component factors have undergone extensive validation work and overall concurrent validity with measures, such as the Cantril Ladder (Cantril, 1965) and the Positive Affect subscale of the Positive and Negative Affect Schedule (PANAS; Watson et al., 1988). In addition, longitudinal measurement fitness and external validity were previously investigated and supported for the items to discriminate between levels of low flourishing (or languishing), moderate, and high levels of flourishing (e.g., Keyes et al., 2020). Moreover, a 38-nation study confirmed the properties, structure, and cross-cultural applicability of the MHC-SF with 8,066 university students (Żemojtel-Piotrowska et al., 2018). Items cover the degree that one possesses *Emotional Well-being* in terms of being “happy, interested in life, or satisfied,” *Social Well-being* as feeling that “one is contributing something worthwhile to society and belonging to the community,” and *Psychological Well-being* as “liking the majority of one’s personality, being capable of managing daily responsibilities, and having warm and trusting relationships with others.” Participants responded to a six-point Likert scale that ranges from 1 (*Never*) to 6 (*Every day*).

### Procedures

The sample was undergraduate students from the Faculties of Arts and Social Sciences in the University of Cape Coast, Ghana. The students were reached at strategic locations, such as lecture theaters, departmental meetings, and halls of residence in the university. The researcher in charge of data collection (D.D.) employed multiple data collection techniques (purposive, accidental, and convenient) sampling approaches to reach the students.

The rationale for inclusion criteria for analysis was for students within the age range of young and emerging adults. As a part of data cleaning, data belonging to 13 participants (six participants, aged 16years and below and the remaining seven participants, 28years and above) were considered as outliers and deleted from the dataset. The resultant age range was 17–27, with a median age of 20 and a mean age of 19.97 and standard deviation of 1.93. About 2% of the remaining 710 participants had missing responses on at least one study variable.

As part of a cross-national project (Wiium and Dimitrova, 2019), the ethical protocol of the current study was approved by the NSD – Norwegian Centre for Research Data. Before administering the questionnaires, the students were briefed about the content of the questionnaire and their verbal consent was first sought. All participants gave their informed consent to participate and allowed the use of their data for analysis.

### Analytical Plan

To assess the pattern of responses of PYD and the MHC among participants, descriptive analyses were undertaken on the demographic variables. Multiple forms of reliability were estimated in keeping with best practices (Dunn et al., 2014). Confirmatory factor analysis (CFA), as an approach to providing measurement models that systematically examine the structural validity of latent constructs (Brown, 2015), was used to confirm the factor structures of the MHC and PYD for further analysis. Finally, structural equation modeling (SEM), as a regression-based family of techniques that allow for standardized procedures for controlling covariates with multiple predictor and outcome variables (e.g., Kline, 2016), was conducted to simultaneously estimate and examine the relations between the 5Cs of PYD and the MHC factors. In line with quantitative research reporting standards (Appelbaum et al., 2018), several indices of model fit were considered, namely, the chi-square (*χ*^2^), Comparative Fit Index (CFI), Tucker-Lewis Index (TLI), Goodness of Fit Index (GFI), Standardized Root Mean Square Residual (SRMR), and Root Mean Square Error of Approximation (RMSEA).

## Results

### Sample Characteristics and Reliability Analysis

Results for the sample characteristics and reliability estimates are summarized in Table 1. Estimates for the study variables among the sample of the 710 university students were calculated in *R* (Version 4.0.3; RStudio Server IDE version 1.3.1093, R Core Team, 2020). Cronbach’s α (*psychometric* package) and McDonald’s *ω* (*MBESS* package) values were greater than 0.7 per conventional guidelines in support for reliability for the MHC factors (MHC-Emotional: *α*=0.81; *ω*=0.81; MHC-Social: *α*=0.82; *ω*=0.82; and MHC-Psychological: *α*=0.87; *ω*=0.87) and the 5Cs of PYD (Competence: *α*=0.71; *ω*=0.72; Confidence: *α*=0.89; *ω*=0.89; Character: *α*=0.84; *ω*=0.84; Caring: *α*=0.83; *ω*=0.84; and Connection: *α*=0.86; *ω*=0.86).

**Table 1:** Demographic and descriptive information for the study variables among youth and emerging adults in Ghana.

Study variable	Youth and emerging adults in Ghana
Gender (*N*, % Female)	710 (51%)
Female	369
Male	354
Age (*M*, *SD*)	19.97 (1.93)
Five Cs of PYD (*M*, *SD*)	
Character (eight items; *α*=0.84)	4.04 (0.77)
Confidence (six items; *α*=0.89)	4.15 (0.85)
Connection (eight items; *α*=0.84)	3.89 (0.75)
Caring (six items; *α*=0.83)	4.02 (0.87)
Competence (six items; *α*=0.71)	3.50 (0.75)
Mental Health Continuum Factors (*M*, *SD*)	
Emotional Well-being (three items; *α*=0.81)	4.69 (1.09)
Social Well-being (five items; *α*=0.82)	4.29 (1.17)
Psychological Well-being (six items; *α*=0.87)	4.81 (1.04)

Response categories for the PYD factors ranged from 1 to 5; MHC factors ranged from 1 to 6.

### Confirmatory Factor Analysis

Structural validity was evaluated using the *lavaan* package in *R* (Rosseel, 2012; version 0.6–7, Rosseel et al., 2020). The robust maximum likelihood estimator was conducted as in prior studies of the scales, and model selection involved the comparison of fit indices and information criteria. Model fit for CFA and SEM was assessed in terms of Chi-square tests, the Tucker-Lewis Index (TLI; acceptable >0.90), the Root Mean Square Error of Approximation (RMSEA; acceptable <0.08), and the Comparative Fit Index (CFI; acceptable >0.90, Brown, 2015).

#### The Five-Factor Model for the 5Cs of Positive Youth Development

Prior to the SEM analysis, the factor structure of the 5Cs of PYD was examined *via* CFA with the model selection procedure depicted in Table 2. An initial CFA of the items in a five-factor model indicated poor model fit: *χ*^2^ (517)=3226.624, *p*=0.000; RMSEA=0.098 (90% *CI*: 0.095–0.101); CFI=0.754; TLI=0.733. As occurred in a prior study of the model with youth in Ghana (Kabir and Wiium, 2021), 14 pairs of same-facet items (in *Character*, *Confidence*, *Connection*, and *Competence*) were allowed to correlate in a model with residual covariances. An examination of the modification indices (MI) revealed cross-loadings of two items (i.e., conduct behavior items reflecting *Character* with high MI values). These items were removed from further CFA analysis. In addition, the modification indices suggested correlations among four pairs of same-construct items, (one pair for *Caring* and the other three for *Confidence*). These pairs of same-construct items were allowed to correlate in a second CFA that suggested adequate model fit: Robust *χ*^2^ (342)=1040.362, *p*=0.000; RMSEA=0.061 (90% *CI*: 0.058–0.065); CFI=0.923; TLI=0.908; SRMR=0.054. Details of the factor loadings and intercorrelations among the latent PYD variables are presented in Table 3, where the majority of loadings ranged from 0.30 to 0.87, suggesting factor determinacy, with the exception of *Competence*, which had two low loadings (0.14–0.24).

**Table 2:** Model comparison of factor structures and psychometric properties for the study instruments.

Model		*df*	Robust minimum function test statistic (*χ*^2^)	*χ*^2^ *p*	Robust CFI	Robust TLI	SRMR	RMSEA (CI)
Positive Youth Development	1-factor model	527	4,848.580	0.000	0.597	0.571	0.094	0.124 (0.121–0.126)
	5-factor model	517	3,226.624	0.000	0.754	0.733	0.082	0.098 (0.095–0.101)
	5-factor model with residual covariances	342	1,040.362	0.000	0.923	0.908	0.054	0.061 (0.058–0.065)
Mental Health Continuum	1-factor model	77	739.623	0.000	0.803	0.767	0.074	0.136 (0.129–0.143)
	3-factor model	74	404.621	0.000	0.904	0.882	0.056	0.098 (0.090–0.105)
	3-factor model with residual covariances	71	303.216	0.000	0.934	0.916	0.045	0.084 (0.076–0.092)

The values for the test and fit statistics are reported for the results of the model with the robust maximum likelihood estimator. CFI, comparative fit index; TLI, Tucker-Lewis index; SRMR, standardized root mean square residual; RMSEA, root mean square error of approximation.

**Table 3:** Summary of the confirmatory factor analysis for the best fitting model of the 5 Cs of Positive Youth Development – factor loadings and correlations.

5 Cs of PYD	Factor loadings range	1	2	3	4
1. Competence	0.14–0.74	-			
2. Confidence^a^	0.71–0.79	0.95	-		
3. Character^b^	0.48–0.87	0.51	0.60	-	
4. Caring	0.30–0.89	0.52	0.54	0.73	-
5. Connection	0.44–0.78	0.62	0.64	0.72	0.68

Model Fit Indices, *χ*^2^ (*df*)=1040.362 (342); RMSEA (90% CI)=0.061 (0.058–0.065); CFI/TLI=0.923/0.908.

a Two items were removed from the 6-item scale due to cross-loadings.

b Three items were removed from the 8-item scale due to cross-loadings.

#### Three-Factor Model of the Mental Health Continuum-Short Form

The procedures for model estimation and selection for the MHC-SF was conducted in keeping with item assignments advised by previous validation studies (Lamers et al., 2011). The results of model comparison procedures are available in Table 2. As a result, a three-factor model with within-factor residual covariances for two similar facet pairs of *Social Well-being* (items 5 with 6 and 7 with 8) and one similar facet pair for *Psychological Well-being* (item 13 with 14) was supported as: Robust *χ*^2^ (71)=303.216, *p*=0.000; RMSEA=0.084 (90% *CI*: 0.076–0.092); CFI=0.934; TLI=0.916; SRMR=0.045. The best fitting model intercorrelations and factor loading ranges are provided in Table 4, which suggested determinacy for the factors (0.61–0.84). The CFA results were comparable to a previous ESEM-based validation of the MHC in Ghana (CFI=0.997, RMSEA=0.024; reported in Appiah et al., 2020b).

**Table 4:** Summary of the confirmatory factor analysis for the best fitting model of the Mental Health Continuum – factor loadings and correlations.

MHC factor	Factor loadings range	1	2	3
1. Emotion	0.72–0.84	-		
2. Social	0.61–0.76	0.64	-	
3. Psychological	0.66–0.80	0.79	0.78	-

Model Fit Indices, *χ*^2^ (*df*)=303.216 (71); RMSEA (90% CI)=0.084 (0.076–0.092); CFI/TLI=0.934/0.916.

### Structural Equation Modeling

Structural equation modeling was carried out in *lavaan* with the robust ML estimator to examine the relations between the best-performing model of the 5Cs of PYD and best-performing three-factor model of the MHC. The demographic variables of age and gender were treated as control variables, as they have been found to influence PYD indicators (Wiium et al., 2018). Acceptable model fit was determined from a combined consideration of the incremental (CFI and TLI), absolute (SRMR), and parsimonious fit indices (RMSEA), such that CFI and TLI values approached 0.90, SRMR values less than or close to 0.06, and RMSEA values were close to or less than 0.08 (Brown, 2015). A supported model was specified with the following results: Robust *χ*^2^ (870)=2323.730, *p*=0.000; RMSEA=0.053 (90% *CI*: 0.051–0.056); CFI=0.899; TLI=0.885; SRMR=0.052. The results of the SEM are available in Table 5. After adjusting for age and gender, standardized coefficients supported paths between *Connection* of PYD and *Emotional Well-being* (*β*=0.39), *Social Well-being* (*β*=0.41), and *Psychological Well-being* (*β*=0.34), and *Caring* and *Psychological Well-being* (*β*=0.27), but not with the PYD factors of *Competence*, *Confidence*, or *Character*. Caution interpreting the results for *Competence* are discussed in the limitations section, but contrary to expectations of convergently positive thriving and flourishing indicators, unsupported negative paths were observed for *Psychological* and *Emotional Well-being*. Similarly, a negative path of low magnitude was observed between *Character* and the MHC factor of *Emotional Well-being* (*β*=−0.14). Paths for *Confidence* were unsupported but strong and positive for *Emotional* and *Psychological Well-being*, but not *Social Well-being*. These results indicate differential contributions for the patterns of PYD-MHC relationships.

**Table 5:** Results of the supported structural equation model for relationships between the 5 Cs of PYD and well-being factors from the Mental Health Continuum among the sample of university students in Ghana (*N*=710).

	Mental health continuum factors		
	Emotional	Social	Psychological
Demographics			
Age	−0.03 (0.02)	−0.01 (0.02)	0.01 (0.02)
Gender	−0.11 (0.07)	−0.06 (0.08)	−0.01 (0.06)
5Cs of PYD^a^			
Competence	−0.74 (1.38)	1.17 (1.42)	−0.24 (0.35)
Confidence	0.79 (0.57)	−0.03 (0.56)	0.84 (0.45) *p* =0.06
Character	−0.14 (0.13)	−0.01 (0.12)	−0.08 (0.11)
Caring	0.11 (0.12)	0.03 (0.12)	0.27 (0.10)^**^
Connection	0.39 (0.11)^***^	0.41 (0.13)^**^	0.34 (0.09)^***^

Model Fit Indices, *χ*^2^ (df)=2323.730 (870); RMSEA (90% CI)=0.053 (0.051–0.056); CFI/TLI=0.899/0.885.

a Standardized coefficient results controlling for age and gender.

** *p*<0.001;

*** *p*<0.0001.

## Discussion

This paper set out to examine relationships between thriving (5Cs of PYD) and flourishing indicators from the mental health continuum (MHC) for youth and emerging adults in Ghana. We modeled relationships between the 5Cs of PYD and three factors of the MHC to sharpen the focus of program efforts and to offer insights on patterns of the multidimensional and culturally shaped nature of well-being in Ghana.

**Figure 1:**
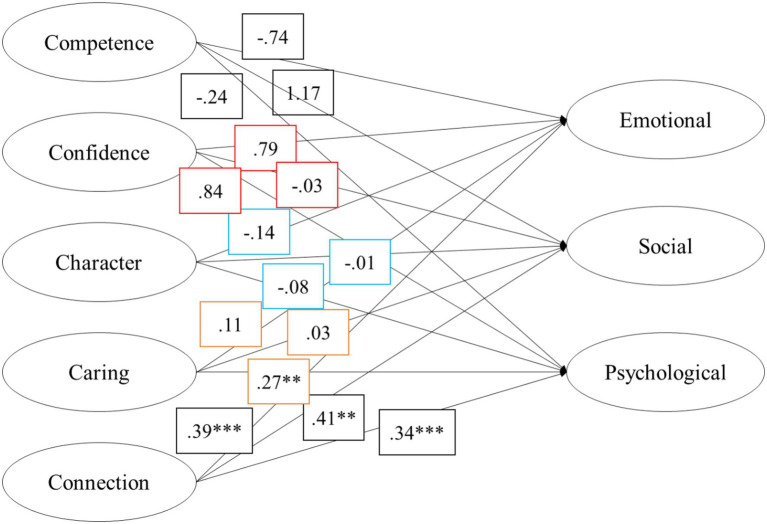
Visual representation of the structural equation model estimating the relations between the 5Cs of PYD and the MHC factors. Model Fit Indices, *χ*^2^ (*df*)=2323.730 (870); RMSEA (90% CI)=0.053 (0.051–0.056); CFI/TLI=0.899/0.885; a standardized coefficient results controlling for age and gender. **p<0.001; ***p<0.0001.

The mean scores of the PYD factors investigated in this study were moderately high and within the ranges observed by prior studies in Ghana on the 5Cs of PYD. Mean factor scores for the MHC were similar to the ranges of previous studies in the context of rural Ghana (Appiah et al., 2020b), and comparable to a representative panel of Dutch participants (Lamers et al., 2011), but were numerically higher than those reported in a prior study of Dutch adolescents, especially for *Social Well-being* (Luijten et al., 2019). All examined factors were reliable in terms of psychometric quality, with the relative exception of *Competence* (see Limitations), as shown in Table 1. Notably, omega coefficients for *Psychological Well-being* were higher in this study (*ω*=0.87) compared to those reported in Appiah et al. (2020b) for rural adults in Ghana (*ω*=0.41). Examinations of the factor structure for the PYD and MHC frameworks (Tables 2–4) were specifiable but found to perform best in models that estimated residual covariances, taking requisite considerations for facets that clearly share overlapping item content (Table 5). Figure 1 depicts the final model for analysis and comparison of constructs.

For our first research question, we expected that the PYD domains would predict positive mental health (Hypothesis 1). For our second research question, our expectations were that *Confidence* would associate strongly with *Psychological Well-being* (Hypothesis 2), and *Character* and *Caring* would be associated with all types of well-being (Hypothesis 3). Additionally, underscoring the role of social relations indicated by cultural factors and prior studies, the components of the 5Cs that handle social competence (*Competence* and *Connection*) and the MHC-SF factor of *Social Well-being* were expected to associate in a positive manner (Hypothesis 4). As depicted in Figure 1, the results suggested that our first hypothesis was supported but the latter was not supported in the *a priori* patterns we anticipated (with the exception of *Connection* and *Social Well-being* and *Caring* and *Psychological Well-being* as one of the MHC factors), as described in the next sections.

Contrary to expectations, the results of the SEM did not support structured relationships between *Confidence*, *Competence*, and *Character* as readily as the factors of *Caring* and *Connection*. One reason for surprise at the unsupported paths was the fact that *Confidence* and *Competence* were strong predictors of environmental attitudes in previous study (Kabir and Wiium, 2021). Given the qualitative cultural model of “moral living” that would support a path for *Character* to *Social Well-being* (Osei-Tutu et al., 2020), and the highly spiritual and religious behavior exhibited in Ghanaian communities (Avogo, 2013), the lack of relationships for *Character* was also unexpected. One explanation for this might be that *Confidence*, *Competence*, and *Character* share a level of self-focus that make them less relevant or available to well-being in the Ghanaian context which is marked by emphasis on relationships and interconnectedness (Wissing et al., 2020). As depicted in Table 5 and Figure 1, the direction and magnitude of the relationships between factors differed meaningfully between frameworks. As a matter of attenuated specificity for the factors, the key findings of our structural model showed supported paths for *Connection*, which was associated with all of the MHC factors, *Emotional Well-being*, *Social Well-being*, and *Psychological Well-being*, and for *Caring*, which was associated with *Psychological Well-being*.

The finding that *Connection*, or having healthy relationships with friends, family, school, and community, aligned with all three forms of well-being on the mental health continuum especially converges with *interdependent* well-being concepts, demonstrated in the preponderance of research on elements of social participation, civic engagement, and micro-level predictors of well-being in Ghana (Avogo, 2013; Addai et al., 2014; Homan, 2017; Amoah, 2018). Adaptive developmental regulation for the youth in our sample appears to reflect relational routes to positive mental health. It also appears consistent with a holistic and possibly non-separate pattern of linkages between eudaimonic and hedonic orientations to well-being in these contexts (Fadiji et al., 2019). Moreover, the finding links to others observed for civic engagement as a route to well-being in other contexts (Wray-Lake et al., 2019), and a similar path that was observed in another sample of Ghanaian youth predicting environmental concerns about increasing pollution (Kabir and Wiium, 2021). This key interpretation of the findings for the emergent paths between thriving and flourishing indicators reflects the value of social relations for maintaining mental health, akin to Ryff’s factor of “positive relations with others” and Osei-Tutu and colleagues’ categorical description of the role for “proper relationality” in a local model of well-being in Ghana (Osei-Tutu et al., 2020). In terms of *Caring* and adaptive developmental regulation, cumulatively, this might indicate that empathic elements of the development of psychological well-being tie to self-views of thriving, perhaps as a matter of empathy-related responding in self-regulation (Eisenberg et al., 2010).

*Competence*, *Confidence*, and *Character* are personal characteristics that are important in other ways, but our lack of structured relationships suggests a lack of the interpersonal relations that are significant for mental health. *Connection* and *Caring* are highly related to the brotherhood and sisterhood perspective that typifies collectivistic cultures and coalesces in the cultural shaping of the psychological makeup of individuals in Ghana. On the one hand, the observed relationships suggest that, through *Connection* and *Caring*, people can give or receive the social support they need to withstand stress and other challenges, which eventually leads to enhanced mental health. This unique pattern of factors is supported by the findings of Homan (2017) who examined the training, competencies, motivation, and readiness of adults delivering strategic interventions by 4-H Ghana, to which having caring volunteers and club leaders was thought to influence the personality development of youth and their willingness to participate in civic processes. On the other hand, Amoah (2018) observed a negative indirect effect of volunteer activities that suggested that social participation is not always favorable as an effect to well-being. This might imply that the qualitatively asserted “*proper* relationality,” a key factor in emic well-being (Osei-Tutu et al., 2020) described in Ghanaian languages, might be situated within this relationship. Also, *Connection* and *Caring* offer support for the Afrocentric view of relational domains as motivationally salient sources of meaning, both for close relationships and community and societal relationships, possibly related to motives for harmonious or better interpersonal relations (Wissing et al., 2020).

## Limitations

This study has limitations chiefly related to the confirmatory model estimated for the 5Cs of PYD with students in Ghana. As discussed in the model selection section, we encountered issues with specifying models, requiring the inclusion of residual correlations to achieve acceptable fit and convergence. Outside of the known issue of cross-loadings for conduct behavior in *Character*, the factor of *Competence*, with its lower reliability and lower item loading observed in the present study, and *Confidence*, with its multiple contributions to model modification, appears to be the source of model identification issues with students in Ghana. This is one of the few times the 5Cs model of PYD has led to estimation issues in the Ghanaian youth context requiring possibly idiosyncratic model modification (Kabir and Wiium, 2021) and follows a pattern that has been reported by Chen et al. (2018), in the form of observing of an inordinately high intercorrelation between *Confidence* and *Competence*.

As noted by Chen et al. (2018), who employed exploratory SEM to limit overestimation of factor correlations typical to conventional CFA, the domain general operationalization of *Confidence* (e.g., akin to self-esteem as a self-view) relative to the domain specific operationalization of *Competence* (e.g., akin to self-efficacy as a self-view) is a plausible diagnosis (e.g., Swann et al., 2007). This suggests that the factors might exhibit a form of nested structure that requires further psychometric investigation, possibly considering a circumplex structure for the two factors, hierarchical modeling opportunities, or insights from other measurement approaches, such as item response theory. On the other hand, we cannot rule out the possibility that simple issues with sample variability could be responsible, as a relatively smaller intercorrelation for the two factors was observed in a previous study with the same items and estimation procedure in a different draw of 995 youth and emerging adults in Ghana from three university sampling sites (Kabir and Wiium, 2021). Considering this, we urge readers to interpret the results of the two factors with caution for the time being, as further psychometric investigations are planned for future research.

## Implications for Further Research and Practice

The present study could pave the way for further research on PYD constructs and other positive developmental indicators to investigate factors that promote positive mental health among youth in Ghana and other African countries. For practitioners, our finding that *Connection* associates with all MHC factors accords with the *community-based* approach used in programs like the multicomponent intervention ILP. In addition to other outcomes, the ILP program showed moderate to large effect sizes in changes for all three MHC factors over time, especially *Emotional Well-being*, among program participants relative to a control group in rural communities of Ghana (Appiah et al., 2020b). In semi-structured interviews with 18 participants by Appiah et al. (2021b), the ILP also provided qualitative themes for well-being in the form of enhanced social networks and relationships and increased vocational productiveness and goal attainment. At the time of hypothesis formation and analysis, these studies were unknown to us, however, based on an inference from the role of *Connection* to all forms of positive mental health measured by the MHC and cumulative synthesis with their careful observations in an implementation context; youth dwelling in urban areas might exhibit similar benefits to well-being in a community-based intervention, such as the ILP, as a clear and plausible way to match PYD principles to interventions (Eichas et al., 2019). Future researchers might implement, extend, or apply the context-specific positive intervention sessions of the ILP for youth in Ghana in addition to those dwelling in rural and poor communities. While more research is needed to disambiguate or determine the thresholds of social support, participation, or relations that are relevant to potentially implicated factors of interdependent well-being, our findings support the notion that emphasizing *Connection* through healthy relationships might be useful for mental health promotion. Building on projects like the ILP with the MHC in the Ghanaian context, incorporating PYD principles in interventions may not only promote thriving but may also help to further enhance positive mental health of youth and emerging adults in Ghana, a step which is important to ensure their contribution to their self-concept and to societal and national development.

## Conclusion

The present study suggests that *Connection* and *Caring* both point to routes of well-being that are important predictors for maintaining flourishing among youthful populations in the context of Ghana. The strongest pattern of path coefficients emerged from the modeling of *Connection* on the MHC continuum, as this factor predicted all three types, suggesting convergence with notions of collective self-efficacy as a path for Ghanaian youth to be visible by people and institutions and develop with a sense of safety. This favors interdependent notions of well-being indicated in findings on social participation, spiritual activities, civic engagement, and theories of interpersonal motives. It also accords with the results of community-based multicomponent positive intervention for older adult residents of poor and rural communities. In sum, our findings indicated that feeling attached and seen within a community and exhibiting concern for others are attributes to follow for young Ghanaian individuals who might flourish as they take on the mantle of contributing to societal development. We hope that this report stimulates further research on well-being and mental health promotion for the up-and-coming generation settling into adulthood in an enterprising Ghana.

## Data Availability Statement

The raw data supporting the conclusions of this article will be made available by the authors, without undue reservation.

## Ethics Statement

The studies involving human participants were reviewed and approved by the NSD – Norwegian Centre for Research Data. The patients/participants provided their written informed consent to participate in this study.

## Author Contributions

RK contributed to original draft writing, methodology, software, formal analysis, and revisions. DD contributed to data collection. NW contributed to original draft writing, conceptualization, methodology, project administration, funding acquisition, software, formal analysis, and revisions. All authors contributed to the article and approved the submitted version.

## Funding

This research was supported by the Faculty of Psychology, University of Bergen, Norway.

## Conflict of Interest

The authors declare that the research was conducted in the absence of any commercial or financial relationships that could be construed as a potential conflict of interest.

## Publisher’s Note

All claims expressed in this article are solely those of the authors and do not necessarily represent those of their affiliated organizations, or those of the publisher, the editors and the reviewers. Any product that may be evaluated in this article, or claim that may be made by its manufacturer, is not guaranteed or endorsed by the publisher.
